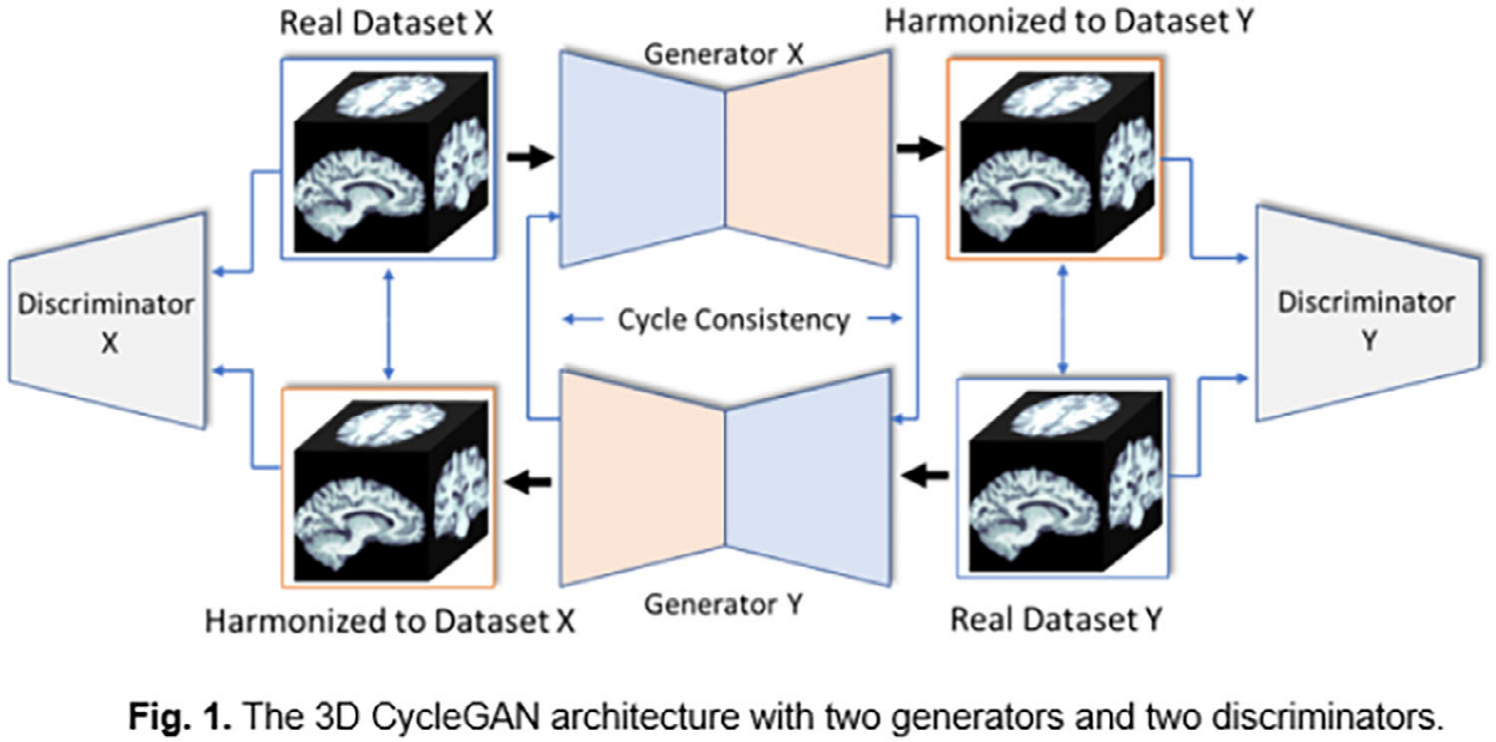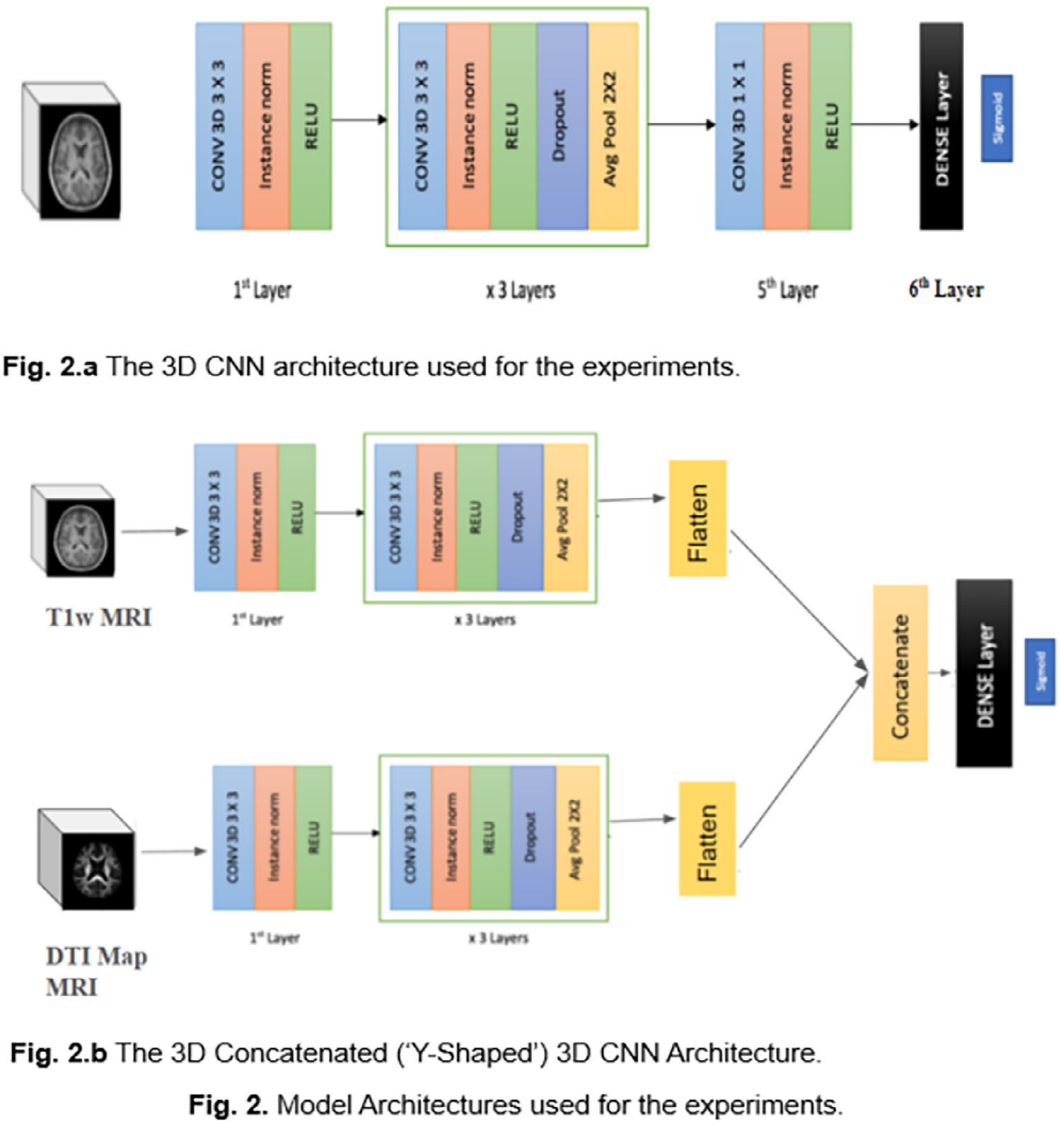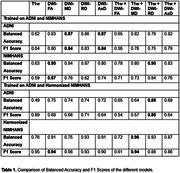# Deep Learning Algorithms for Alzheimer’s Disease Detection based on Diffusion MRI: Tests in Indian and North American Cohorts

**DOI:** 10.1002/alz.089294

**Published:** 2025-01-09

**Authors:** Tamoghna Chattopadhyay, Neha Ann Joshy, Saket S. Ozarkar, Ketaki S. Buwa, Yixue Feng, Emily Laltoo, Sophia I Thomopoulos, Julio E Villalon‐Reina, Himanshu Joshi, Ganesan Venkatasubramanian, John P John, Paul M. Thompson

**Affiliations:** ^1^ Imaging Genetics Center, Mark and Mary Stevens Neuroimaging & Informatics Institute, Keck School of Medicine, University of Southern California, Marina del Rey, CA USA; ^2^ Imaging Genetics Center, Mark and Mary Stevens Neuroimaging & Informatics Institute, University of Southern California, Marina del Rey, CA USA; ^3^ Multimodal Brain Image Analysis Laboratory, National Institute of Mental Health and Neurosciences (NIMHANS), Bengaluru, Karnataka India; ^4^ Translational Psychiatry Laboratory, National Institute of Mental Health and Neurosciences (NIMHANS), Bengaluru, Karnataka India; ^5^ Multimodal Brain Image Analysis Laboratory, National Institute of Mental Health and Neurosciences (NIMHANS), Bengaluru India

## Abstract

**Background:**

Deep learning models based on convolutional neural networks (CNNs) have been used to classify Alzheimer’s disease or infer dementia severity from T1‐weighted brain MRI. Here we tested the added value of incorporating information from 3D diffusion‐weighted MRI ‐ a technique sensitive to microstructural differences ‐ alongside traditional 3D T1‐weighted images. We evaluated our classifier’s performance on cohorts from India and North America. We also tested if classification accuracy improved after applying 3D CycleGAN approach to harmonize the imaging datasets prior to training the CNN models. Our experiments reveal that, in the majority of cases, classification performance sees improvement following harmonization, particularly when utilizing dMRI as input.

**Method:**

We analyzed data from 1,195 participants (age: 74.36 ±7.74 years; 600 F/595 M; 633 CN/421 MCI/141 dementia), who had both structural T1w and dMRI, from the Alzheimer's Disease Neuroimaging Initiative (ADNI) dataset. The second dataset came from an Indian population assessed at NIMHANS in Bangalore, India – a population not typically well represented in neuroimaging studies. It had 301 participants (age: 67.23 ±7.86 years; 169 F/132 M) with a distribution of (123 CN/88 MCI/90 dementia). We used the T1ws and dMRI‐derived maps of microstructural parameters ‐ FA, MD, RD and AxD as inputs to our model. Initially, a 3D CycleGAN (Figure 1) was used to harmonize the two datasets. Then, both a 3D CNN architecture (Figure 2a) and a Y‐shaped 3D CNN architecture (Figure 2b) were used for the classification task. The latter can take dual modalities as input simultaneously. Performance was assessed using Balanced Accuracy and F1 Score.

**Result:**

Table 1 shows the performance of the models. The best performances are seen for the Y‐shaped model, and when harmonization is used before training.

**Conclusion:**

In general, performance improved after harmonizing the datasets using the unsupervised 3D CycleGAN architecture. Training the model on the merged dataset incorporating diverse cohorts made the classifier more robust. When smaller datasets are available for training, AD classification was more accurate when based on DWI‐derived maps, compared to T1w images. A concatenated model incorporating multiple image modalities outperformed a model using only T1w MRIs as input.